# Supplementary material for: 5'-UTR SNP of FGF13 causes translational defect and intellectual disability
Source: eLife. 2021 Jun 29;10:e63021. doi: 10.7554/eLife.63021 (PMC8241442; doi:10.7554/eLife.63021)
Supplement: Supplementary file 1. [file elife-63021-supp1.docx]

| **Supplementary File 1. List of 262 reported ID related genes from database and literature screening** | | | | | | | | | |
| --- | --- | --- | --- | --- | --- | --- | --- | --- | --- |
| **Gene name** | | | | | | | | | |
| *ABCD1* | *CDH9* | *DKC1* | *FOXG1* | *HSD17B10* | *MBTPS2* | *NXF5* | *RELN* | *SLC6A3* | *SYT1* |
| *ACSL4* | *CDK5* | *DLG2* | *FSTL1* | *HUWE1* | *MECP2* | *OAT* | *RET* | *SLC6A6* | *SYT4* |
| *ADAM9* | *CDKL5* | *DLG3* | *FTSJ1* | *IDS* | *MED12* | *OCRL* | *ROCK1* | *SLC6A7* | *SYT5* |
| *AFF2* | *CHD8* | *DLG4* | *FUS* | *IGBP1* | *MID1* | *OFD1* | *RPL10* | *SLC6A8* | *TARDBP* |
| *AGTR2* | *CHRNA1* | *DLGAP2* | *GAD1* | *IKBKG* | *MTM1* | *OLIG2* | *RPS6KA3* | *SLC6A9* | *TH* |
| *ALS2* | *CKB* | *DMD* | *GAD2* | *IL1RAPL1* | *NAA10* | *OPHN1* | *SHANK1* | *SLC9A6* | *THOC2* |
| *AP1S2* | *CKM* | *DRD1* | *GAMT* | *IQSEC2* | *NDEL1* | *OTC* | *SHANK2* | *SLIT2* | *TIMM8A* |
| *APP* | *CKMT1A* | *DRD2* | *GATM* | *ISL1* | *NDP* | *OXT* | *SHANK3* | *SMAD4* | *TLX3* |
| *ARG1* | *CKMT1B* | *DVL1* | *GDI1* | *ISL2* | *NDUFA1* | *PAFAH1B1* | *SHH* | *SMC1A* | *TSPAN7* |
| *ARG2* | *CKMT2* | *DYRK1A* | *GK* | *JAK2* | *NEUROD2* | *PAK3* | *SHROOM4* | *SMO* | *UBE2A* |
| *ARHGEF6* | *CLCN4* | *EFNA2* | *GPC3* | *KDM5C* | *NHS* | *PARK2* | *SLC16A2* | *SMS* | *UBE3A* |
| *ARHGEF9* | *CNTF* | *EIF2S3* | *GRIA2* | *KIAA2022* | *NLGN1* | *PAX7* | *SLC17A7* | *SNAP25* | *UPF3B* |
| *ARX* | *CNTN2* | *ELFN1* | *GRIA3* | *KLF8* | *NLGN2* | *PCDH19* | *SLC17A8* | *SOD1* | *WNT3A* |
| *ASCL1* | *CNTNAP1* | *ELFN2* | *GRIK2* | *KRAS* | *NLGN3* | *PDHA1* | *SLC18A1* | *SOX2* | *ZDHHC15* |
| *ASL* | *CNTNAP2* | *EN1* | *GRIN2A* | *LAMP2* | *NLGN4X* | *PGK1* | *SLC18A2* | *SOX3* | *ZDHHC9* |
| *ASS1* | *CNTNAP3* | *EN2* | *GRIN2B* | *LAS1L* | *NOS1* | *PHF6* | *SLC1A3* | *SRGAP1* | *ZNF41* |
| *ATP6AP2* | *CNTNAP3B* | *EPHA7* | *GRM5* | *LHX6* | *NOTCH1* | *PHF8* | *SLC32A1* | *SRGAP2* | *ZNF674* |
| *ATP7A* | *CNTNAP4* | *ESR1* | *HCCS* | *LPHN2* | *NOTCH3* | *PLP1* | *SLC6A1* | *SRGAP3* | *ZNF711* |
| *ATRX* | *CRH* | *ESR2* | *HCFC1* | *LPHN3* | *NPAS3* | *PORCN* | *SLC6A11* | *SRPX2* | *ZNF81* |
| *AVP* | *CTNNB1* | *FANCB* | *HDAC6* | *LRRTM1* | *NPAS4* | *PQBP1* | *SLC6A12* | *SS18L1* |  |
| *BCL2* | *CUL4B* | *FGD1* | *HDAC8* | *LRRTM2* | *NRAS* | *PRPS1* | *SLC6A13* | *STX1A* |  |
| *BCL2L1* | *CYFIP1* | *FGF13* | *HES1* | *LRRTM3* | *NRCAM* | *PSEN1* | *SLC6A14* | *STX3* |  |
| *BCOR* | *CYFIP2* | *FLNA* | *HES3* | *LRRTM4* | *NRXN1* | *PTEN* | *SLC6A15* | *STX4* |  |
| *BDNF* | *DCC* | *FLRT1* | *HES5* | *MAGT1* | *NRXN2* | *RAB39B* | *SLC6A18* | *SYN1* |  |
| *BRWD3* | *DCLK1* | *FLRT2* | *HOMER1* | *MAOA* | *NRXN3* | *RAB40AL* | *SLC6A19* | *SYNGAP1* |  |
| *CASK* | *DCX* | *FLRT3* | *HOMER2* | *MBD3* | *NSDHL* | *RAPSN* | *SLC6A2* | *SYNGR1* |  |
| *CDH10* | *DISC1* | *FMR1* | *HPRT1* | *MBD5* | *NUMBL* | *RBM10* | *SLC6A20* | *SYP* |  |
