# Supplementary material for: 5'-UTR SNP of FGF13 causes translational defect and intellectual disability
Source: eLife. 2021 Jun 29;10:e63021. doi: 10.7554/eLife.63021 (PMC8241442; doi:10.7554/eLife.63021)
Supplement: Supplementary file 2. [file elife-63021-supp2.docx]

| **Supplementary File 2. List of SNPs and In-Dels of three families from exon-capture sequencing** | | | | | | | |
| --- | --- | --- | --- | --- | --- | --- | --- |
|  | | **SNP** | | | **In-Dels** | | |
| **Subject** | | **Located on ID-related genes** | **Predicted as pathogenic by clinVar** | **Pathogenic mutations** | **Located on ID-related genes** | **Predicted as pathogenic by clinVar** | **Pathogenic mutations** |
| Family 1 | Father | 856 | 1 | 1 | 466 | 0 | 0 |
|  | Mother | 1235 | 1 | 1 | 549 | 0 | 0 |
|  | Son | 1096 | 0 | 0 | 527 | 0 | 0 |
| Family 2 | Father | 1189 | 0 | 0 | 599 | 0 | 0 |
|  | Mother | 1407 | 0 | 0 | 578 | 0 | 0 |
|  | Son | 1186 | 0 | 0 | 541 | 0 | 0 |
| Family 3 | Father | 1195 | 0 | 0 | 537 | 0 | 0 |
|  | Mother | 1195 | 0 | 0 | 543 | 0 | 0 |
|  | Son | 1195 | 0 | 0 | 535 | 0 | 0 |
