# Supplementary material for: 5'-UTR SNP of FGF13 causes translational defect and intellectual disability
Source: eLife. 2021 Jun 29;10:e63021. doi: 10.7554/eLife.63021 (PMC8241442; doi:10.7554/eLife.63021)
Supplement: Supplementary file 3. [file elife-63021-supp3.docx]

| **Supplementary File 3. List of SNPs, In-Dels and CNVs of three children from whole genome sequencing** | | | | | | | | | |
| --- | --- | --- | --- | --- | --- | --- | --- | --- | --- |
|  | **SNP** | | | **In-Dels** | | | **CNVs** | | |
| **Subject** | **Located on ID-related genes** | **Predicted as pathogenic by clinVar** | **Pathogenic mutations** | **Located on ID-related genes** | **Predicted as pathogenic by clinVar** | **Pathogenic mutations** | **Located on ID-related genes** | **Predicted as pathogenic by clinVar** | **Pathogenic mutations** |
| Child 1 | 3655 | 0 | 0 | 39 | 0 | 0 | 2 | 30 | 0 |
| Child 2 | 3687 | 1 | 1^a^ | 39 | 0 | 0 | 2 | 29 | 0 |
| Child 3 | 3564 | 0 | 0 | 11 | 0 | 0 | 1 | 27 | 0 |

^a^ One heterozygou nonsynonymous SNP in RAF1 which may cause cardiomyopathy dilated
