# Supplementary material for: 5'-UTR SNP of FGF13 causes translational defect and intellectual disability
Source: eLife. 2021 Jun 29;10:e63021. doi: 10.7554/eLife.63021 (PMC8241442; doi:10.7554/eLife.63021)
Supplement: Supplementary file 4. [file elife-63021-supp4.docx]

| **Supplementary File 4. Clinical records of 3 intellectual disability individuals** | | | | | | | |
| --- | --- | --- | --- | --- | --- | --- | --- |
|  | | **Individual 1** | | **Individual 2** | | **Individual 3** | |
| Gender | | Male | | Male | | Male | |
| Age at visit | | 11 years | | 8 years | | 13 years | |
| School roll | | Grade 6 in special school | | Grade 2 in special school | | Grade 8 in special school | |
| Parental age | | Mother, 40 years | | Mother, 38 years | | Mother, 41 years | |
|  |  | Father, 43 years | | Father, 33 years | | Father, 44 years | |
| The disease diagnosis | | Severe intellectual disability | | Severe intellectual disability | | Severe intellectual disability | |
| **Mutations** | | | | | | | |
| Chromosome position | | chrX: 138,286,301 | | chrX: 138,286,301 | | chrX: 138,286,301 | |
| Base change | | C > G | | C > G | | C > G | |
| **Intelligence and social adaptive capacity tests** | | | | | | | |
| Wechsler Intelligence Scale for Children (WISC) | | Full Scale IQ (FSIQ) | <40 | Full Scale IQ (FSIQ) | <40 | Full Scale IQ (FSIQ) | <40 |
|  |  | Verbal IQ (VIQ) | <40 | Verbal IQ (VIQ) | Not report (NR) | Verbal IQ (VIQ) | 43 |
|  |  | Performance IQ (PIQ) | <40 | Performance IQ (PIQ) | NR | Performance IQ (PIQ) | 55 |
|  |  | General knowledge | 1 | General knowledge | NR | General knowledge | 10 |
|  |  | Similarities | 1 | Similarities |  | Similarities | 4 |
|  |  | Arithmetic | 1 | Arithmetic |  | Arithmetic | 4 |
|  |  | Vocabulary | 1 | Vocabulary |  | Vocabulary | 30 |
|  |  | Comprehension | 1 | Comprehension |  | Comprehension | 14 |
|  |  | Picture Completion | 1 | Picture Completion |  | Picture Completion | 19 |
|  |  | Digit Span | 1 | Digit Span |  | Digit Span | 20 |
|  |  | Block design | 1 | Block design |  | Block design | 6 |
|  |  | Symbol search | 1 | Symbol search |  | Symbol search | 11 |
|  |  | Coding | 1 | Coding |  | Coding | 21 |
| The Vineland Adaptive Behavior Scales (VABS) | | Rank | Profound | Rank | NR | Rank | Mild |
|  |  | Score | 5 | Score | NR | Score | 9 |
| **Birth** | | | | | | | |
| Gravida/para (GP) | | G_2_P_1_ | | G_1_P_1_ | | G_1_P_1_ | |
| Vaginal delivery | | Cesarean section vaginal delivery (premature rupture of the amniotic fluid) | | Spontaneous vaginal delivery | | Cesarean section vaginal delivery (circular of umbilical cord) | |
| Gestation time | | Full term (39-41 weeks) | | Full term (39-41 weeks) | | Full term (39-41 weeks) | |
| Birth weight (g) | | 3450 | | 3950 | | 3400 | |
| Apgar score | | 10 | | 10 | | 10 | |
| Status during pregnancy period | | Mother bleeding on pregnancy-month-2, treated with progesterone. Mother caught a cold on pregnancy-month-4. The first child encountered spontaneous abortion for unexplained reasons. | | No exceptions during pregnancy period. | | Mother fainted on pregnancy-month-5 in the subway for unknown reasons. | |
| History of poisonous and harmful material contacts during pregnancy | | - | | - | | - | |
| **Development** | | | | | | | |
| Summarized history of growth and development | | The motor development of infant was normal, and he could walk on 15 months. The child was hyperactive but quiet. He could not speak until 3 years old. Autism was suspected in the hospital. After given neurotrophic drugs and rehabilitation, the child learned simply "monosyllabic" sounds. He went to the nursery school, but was returned due to the disability of taking care for himself. He went to the special school at 7 years old. | | The motor development of infant was normal, and he could walk on 11 months. The child did not make sounds except crying within 18 months. He could not speak on 3 years old. He was diagnosed as hypophrenia in the hospital, and given rehabilitation training. He did not go to the nursery school, and went to the special school at 7 years old. | | The motor development of infant was normal, and he could walk on 13 months. The child did not make sounds until 2 years old. He can speak simply at 3 years old. Autism was suspected at 5 yers old by the pediatric hospital. He went to the nursery school, and spend first and second grade in the primary school, then transferred to the special school at third grade for halfhearted class attendance and low scores. | |
| **Clinical details** | | | | | | | |
| Bradykinesia | | + | | - | | - | |
| Expressive speech | | Very simple words | | absent | | Normal | |
| Intellectual disability | | Very severe | | severe | | Moderate | |
| Self-care ability | | Defecation needs help | | Urination and defecation needs help | | All self-care | |
| Pain threshold | | High | | Normal | | High | |
| Afraid of strangers | | - | | - | | - | |
| Frequent emesis | | + | | - | | - | |
| Frequent soliloquy | | - | | - | | + | |
| Chronic disease history | | - | | - | | - | |
| **Family history** | | | | | | | |
| Father | Education level | Bachelor degree | | Bachelor degree | | Junior college degree | |
|  | Career | Company founder | | Company clerk | | Company clerk | |
|  | Age when conceive the child | 32 | | 25 | | 31 | |
|  | History of present illness | Have lumbar disease, no chronic illness. | | No chronic illness | | No chronic illness | |
|  | Sib neurological history | No intelligence and mental disorders within 3 generations. The father has a younger brother who has a son without intelligence problems. | | No intelligence and mental disorders within 3 generations. The father was the only child. | | The father has a younger female cousin who has a daughter with intellectual disability (diagnosed as phenylketonuria). Rest members have no intelligence and mental disorders within 3 generations. | |
| Mother | Education level | High school | | Bachelor degree | | Bachelor degree | |
|  | Career | Supermarket clerk | | Financial staff | | High school teacher | |
|  | Age when conceive the child | 29 | | 30 | | 28 | |
|  | History of present illness | No chronic illness | | No chronic illness | | Had hyperthyroidism at 40 years old | |
|  | Sib neurological history | No intelligence and mental disorders within 3 generations. The mother has an elder sister who has a 17-year-old son in high school without apparent intelligence problems. | | No intelligence and mental disorders within 3 generations. The mother has a bachelor-degree younger brother who has a son without apparent intelligence problems. | | No intelligence and mental disorders within 3 generations. The mother has an elder brother who has a normal daughter. The mother also has a younger brother who has a normal daughter. | |
