# Supplementary material for: 5'-UTR SNP of FGF13 causes translational defect and intellectual disability
Source: eLife. 2021 Jun 29;10:e63021. doi: 10.7554/eLife.63021 (PMC8241442; doi:10.7554/eLife.63021)
Supplement: Supplementary file 5. [file elife-63021-supp5.docx]

| **Supplementary File 5. Primers and oligonucleotides used for real-time PCR, Cas9 targeting, sequencing, plasmid construction and biotin probe** | |
| --- | --- |
| Name of Genes for Real-Time PCR | Primers and oligonucleotides (from 5' to 3') |
| *FGF13* | 5'-TCGAGACTTGCCGCCCTAAG-3' |
|  | 5'-GTGGTAGCCTTGTCGGCTGT-3' |
| *GAPDH* | 5'-GACCTGCCGTCTAGAAAAACCT-3' |
|  | 5'-CTGTTGCTGTAGCCAAATTCGT-3' |
| *FGF13*-transcript variants-R-all | 5'-GTGCTGTCCTCATCTTTGGTGC-3' |
| *FGF13*-transcript variant-1-F | 5'-CGACAAAAACAAGTTAAATGTCTT-3' |
| *FGF13*-transcript variant-2/3/4-F | 5'-TGGGATCTATGAGTGGAAAGGT-3' |
| *FGF13*-transcript variant-3-F | 5'-GCTCCTGCTTGCCTTCCTCC-3' |
| *FGF13*-transcript variant-2/3-F | 5'-TGCGGAATTAAAGAAAAAAGAGTC-3' |
| *FGF13*-transcript variant-5-F | 5'-CTCCTTGCCTTCCACCGTGATGT-3' |
| *FGF13*-transcript variant-6-F | 5'-CATGGCTTTGTTAAGGAAGTCGT-3' |
| *NANGO* | 5'-GCAGAAGGCCTCAGCACCTA-3' |
|  | 5'-AGGTTCCCAGTCGGGTTCA-3' |
| *PAX6* | 5'-CTTTGCTTGGGAAATCCGAG-3' |
|  | 5'-AGCCAGGTTGCGAAGAACTC-3' |
| *MAP2* | 5'-TTGGTGCCGAGTGAGAAGAA-3' |
|  | 5'-GGTCTGGCAGTGGTTGGTTAA-3' |
| *GFAP* | 5'-AGGTCCATGTGGAGCTTGAC-3' |
|  | 5'-GCCATTGCCTCATACTGCGT-3' |
| *NES* | 5'-TGCGGGCTACTGAAAAGTTC-3' |
|  | 5'-GGCTGAGGGACATCTTGAG-3' |
| *PTBP1* | 5'-CTTTGCCATTCCTCAAGC-3' |
|  | 5'-CACCGTAGACGCCGAAA-3' |
| *Fgf13* | 5'-TAGGGAGAGAGAGAAAGAGAGC-3' |
|  | 5'-CTTGGTCACCTTTCCACTCAT-3' |
| *Fgf13*-transcript variant1-F | 5'-CCAGAGCCTCAGCTTAAGGGT-3' |
| *Fgf13*-transcript variants-R-all | 5'-CTCAGGTGTGAAATGTTCCGAG-3' |
| *Fgf13*-transcript variant2-F | 5'-CAAGCAAGCTGGGATCTATGAG-3' |
| *Fgf13*-transcript variant4-F | 5'-CACAGGAATACATTATGTTACGAC-3' |
| *Fgf13*-transcript variant3-F | 5'**-**TGTCTGGGTAGGACCATCTTAT-3' |
| *Gapdh* | 5'-GTTGTCTCCTGCGACTTCA-3' |
|  | 5'-GGTCCAGGGTTTCTTACTCC-3' |
| Name of Primers for Cas9 Targeting | Primers and oligonucleotides (from 5' to 3') |
| Control iPSCs Cas9 targeting | 5'-CACCGACAAGGTTCGCTCAGAAGA-3' |
|  | 5'-AAACTCTTCTGAGCGAACCTTGTC-3' |
| SSOD repair template | 5'-AAGCACTCTCCCAAGTCCGACCCGCTCGGCGAGGACTTCCGTCTTGTGAGCGAACCTTGTCAAGCAAGCTGGGATCTATGAGTGGAAAGGT-3' |
| Mouse Cas9 targeting | 5'-CACCCGGCGAGGACTTCCTTCTCC-3' |
|  | 5'-AAACGGAGAAGGAAGTCCTCGCCG-3' |
| Mouse SSOD repair template | 5'-AAGCGCTCTCGCAAGTCCAGCCAGCTCGGCGAGGACTTCCTTCTCGTGGACGAATCTTGTCAAGCAAGCTGGGATCTATGAGTGGAAAGGT-3' |
| Name of Primers for Sequencing | Primers and oligonucleotides (from 5' to 3') |
| hFGF13 point mutation | 5'-CGCCGCTTTGTCTCTTCAC-3' |
|  | 5'-CACGCACACACATGCACTCA-3' |
| iPSCs Cas9 off target-1 | 5'-GTTACTTTACCACTTTGTCCCC-3' |
|  | 5'-CTGAGAAAAAGTGTGAGAGCCT-3' |
| iPSCs Cas9 off target-2 | 5'-ACAGACACTGATTTGATACATAAG-3' |
|  | 5'-TGCCATTCTGCCTTTATTTTCATC-3' |
| iPSCs Cas9 off target-3 | 5'-GGCGAGTTGAATCTGGTGGG-3' |
|  | 5'-GTTCTGGATTTTAGGAGGCTGC-3' |
| iPSCs Cas9 off target-4 | 5'-CGTTCATTGCCATATTTAGGAGC-3' |
|  | 5'-GTGGCCCGGGTCAGGGTTTT-3' |
| iPSCs Cas9 off target-5 | 5'-CCCACGTGTATACTTAATCCTGT-3' |
|  | 5'-CAAGTCCTGGATCAGATTAAAATC-3' |
| iPSCs Cas9 off target-6 | 5'-GGTATAATTGTAGCTGCTAATAAG-3' |
|  | 5'-CTGTTATTAAGTCAAGAGTCAAAG-3' |
| iPSCs Cas9 off target-7 | 5'-GGAACCTAACTAAAATGCTAAGAT-3' |
|  | 5'-AATGAGTGCCGATTTTATTTCACT-3' |
| iPSCs Cas9 off target-8 | 5'-TGGAAGAACACGATGCTGGAC-3' |
|  | 5'-GGGAATAGGCATCTTTGAGGTG-3' |
| iPSCs Cas9 off target-9 | 5'-CACTCCAGTAGGTCCTTTCCAAAG-3' |
|  | 5'-TTTGATTTCCCCATCGAGACTG-3' |
| iPSCs Cas9 off target-10 | 5'-GATAGGTACAGGAGTCCACAG-3' |
|  | 5'-ACTGGAACCTGGAGGGGAAG-3' |
| Mouse FGF13 point mutation | 5'-GGCGGCGACAGCAGCGGC-3' |
|  | 5'-CAAGGGGGCGAGGGCAACCT-3' |
| Mouse Cas9 off target-1 | 5'-GATTGTGAGCCACCATGTAGTT-3' |
|  | 5'-GCTTACTTTCGTGCTCTCTGG-3' |
| Mouse Cas9 off target-2 | 5'-GACCAGATATGGAACTGTGTTGT-3' |
|  | 5'-GGAGATACATCCTCACGGGGT-3' |
| Mouse Cas9 off target-3 | 5'-TACTTTATCACAAGAACTGCTCAT-3' |
|  | 5'-GAAGAGGAACAAGAAACACTCAC-3' |
| Mouse Cas9 off target-4 | 5'-CTCCACTGTATCAAATATGCCTG-3' |
|  | 5'-CCAGAAGTTGCTAATGGAAGAC-3' |
| Mouse Cas9 off target-5 | 5'-CTTCCTGGTTCTCCTGTTGCC-3' |
|  | 5'-GAAAGGGCTTAGAGCATCCTCT-3' |
| Mouse Cas9 off target-6 | 5'-GCCCAGTGCCTGAGAGAAGC-3' |
|  | 5'-TACATACTTGCTTAGCTGCCAG-3' |
| Mouse Cas9 off target-7 | 5'-CCCCAACCATACCCATCCTCT-3' |
|  | 5'-GTTTCTGGCTGTTCTGGGGTT-3' |
| Mouse Cas9 off target-8 | 5'-GAAAGGTTCGACAGGGTCAGG-3' |
|  | 5'-AATTTCTCCACTGACACACTTAC-3' |
| Mouse Cas9 off target-9 | 5'-TGCACAATATAGTTCAGGAACTG-3' |
|  | 5'-GGTCTTCTGGCTTCGGTCCTT-3' |
| Mouse Cas9 off target-10 | 5'-CATCATCTCCCCCTAAACTGG-3' |
|  | 5'-GTCTCCAATACCAGGGTTCTAT-3' |
| Name of Plasmids | Primers and oligonucleotides (from 5' to 3') |
| FGF13 expressing plasmid | 5'-CCGGAATTCGTGGCTCTCTAGGACCGGAG-3' |
|  | 5'-CGCGGATCCACGTTGATTCATTGTGGCTC-3' |
| FGF13 mutation | 5'-GAGGACTTCCGTCTTGTGAGCGAACCTTGTC-3' |
|  | 5'-GACAAGGTTCGCTCACAAGACGGAAGTCCTC-3' |
| FGF13 5'-UTR psiCHECK^TM^-2 | 5'-CTAGCTAGCGTGGCTCTCTAGGACCGGAGA-3' |
|  | 5'-GATGCTAGCATCCCAGCTTGCTTGACAAG-3' |
| mFGF13 expressing plasmid | 5'-CCGGAATTCCTGCCTCTCTGGGATCAGAG-3' |
|  | 5'-CGCGGATCCACGTTGATTCGTTGTGGCT-3' |
| mFGF13 mutation | 5'-GAGGACTTCCTTCTCGTGGACGAATCTTGT-3' |
|  | 5'-ACAAGATTCGTCCACGAGAAGGAAGTCCTC-3' |
| Mouse 5'-UTR psiCHECKTM-2 | 5'-CTAGCTAGCCTGCCTCTCTGGGATCAGAG-3' |
|  | 5'-GATGCTAGCAGATCCCAGCTTGCTTGAC-3' |
| hFGF13-transcript variant-R-all | 5'-GTATCCCTCACTGTTCATTGC-3' |
| hFGF13--transcript variant1-F | 5'-AGGGGCAGCAAAAGAAGCGGT-3' |
| hFGF13-transcript variant2/3/4-F | 5'-AGGACTTCCGTCTTCTGAGCG-3' |
| hFGF13-transcript variant5-F | 5'-GTAACATGTGATTTGCTCCTCCT-3' |
| hFGF13--transcript variant6-F | 5'-TCTGTGTCTGCTCCAAATGTAG-3' |
| mFGF13-transcript variants-R-all | 5'-CTCAGGTGTGAAATGTTCCGAG-3' |
| mFGF13--transcript variant1-F | 5'-CAAGACCAGCTGCGACAAAAAC-3' |
| mFGF13-transcript variant2/4-F | 5'-CAGCGGGGGAAAAAGCGGA-3' |
| mFGF13-transcript variant4-F | 5'-GACGCCCCCCCTGGCACAC-3' |
| mFGF13--transcript variant3-F | 5'-TGTAGACAGCAATTGTCTGGGT-3' |
| IRES 5'-UTR plasmid | 5'-TTTGGCGTCTTCCATAGATCCCAGCTTGCTTGACAAG-3' |
|  | 5'-AATAGCAGCTGACCGGTGGCTCTCTAGGACCGGAG-3' |
| IRES random sequence plasmid | 5'-TTTGGCGTCTTCCATGGTCTGTTGAATGTCGTGAAGG-3' |
|  | 5'-AATAGCAGCTGACCGCACCGAGGCCCCAGATCAG-3' |
| pFastBac1-PTBP1/2 | 5'-CGCGGATCCAACTCCTAAAAAACCGCCACCATGGACGGAATCGTCACTG-3' |
|  | 5'-CCGGAATTCCTTATCGTCGTCATCCTTGTAATCAATTGTTGACTTGGAGAAAGA-3' |
| pcDNA3-Flag-wPUF/mPUF-mPTBP2 | 5'-CGGCAGCGGTAGCTCTAGAATGGACGGAATTGTCACTGAG-3' |
|  | 5'-CATGCTCGAGCGGCCGCTTAGATTGTTGACTTGGAGAAAG-3' |
| Name of RNA Oligonucleotides for Biotin Pull-Down | RNA oligonucleotides (from 5' to 3') |
| WT hFGF13 probe | 5'-biotin-CUUCCGUCUUCUGAGCGCUUCCGUCUUCUGAGCGCUUCCGUCUUCUGAGCG-3' |
| Mut hFGF13 probe | 5'-biotin-CUUCCGUCUUGUGAGCGCUUCCGUCUUGUGAGCGCUUCCGUCUUGUGAGCG-3' |
| WT mFGF13 probe | 5'-biotin-CUUCCUUCUCCUGGACGCUUCCUUCUCCUGGACGCUUCCUUCUCCUGGACG-3' |
| Mut mFGF13 probe | 5'-biotin-CUUCCUUCUCGUGGACGCUUCCUUCUCGUGGACGCUUCCUUCUCGUGGACG-3' |
| Name of siRNA Oligonucleotides | RNA oligonucleotides (from 5' to 3') |
| Human siPTBP1 | 5'-GCACAGUGUUGAAGAUCAUTT-3' |
|  | 5'-AUGAUCUUCAACACUGUGCTT-3' |
| Mouse siPTBP2-1 | 5'-GCCAACGGUAACGAUAGUATT-3' |
|  | 5'-UACUAUCGUUACCGUUGGCTT-3' |
| Mouse siPTBP2-2 | 5'-CCAGUACUCCAAUCACAAATT-3' |
|  | 5'-UUUGUGAUUGGAGUACUGGTT-3' |
| Mouse siPTBP2-3 | 5'-GCAUGUGCAGACUGGUCUATT-3' |
|  | 5'-UAGACCAGUCUGCACAUGCTT-3' |
