# Supplementary material for: 5'-UTR SNP of FGF13 causes translational defect and intellectual disability
Source: eLife. 2021 Jun 29;10:e63021. doi: 10.7554/eLife.63021 (PMC8241442; doi:10.7554/eLife.63021)
Supplement: Supplementary file 6. [file elife-63021-supp6.docx]

| **Supplementary File 6. Antibodies, regents, cell lines, plasmid constructs, softwares and other resources used in this study** | | |
| --- | --- | --- |
| REAGENT or RESOURCE | SOURCE | IDENTIFIER |
| Antibodies | | |
| IB: anti-FGF13 | Santa Cruz | Cat# sc-16811 RRID: AB_2104044 |
| IB: anti-FGF13 | (*Wu et al.*, *2012*) | Home-made by immunization with FGF13B 182-192 aa |
| IB/IP: anti-HA | Sigma | Cat# H3663 RRID: AB_262051 |
| IB: anti-Flag | Sigma | Cat# F3165 RRID: AB_259529 |
| IB: anti-actin | Chemicon | Cat# MAB1501 RRID: AB_2223041 |
| IB: anti-MATR3 | Abcam | Cat# ab151739 RRID:AB_2885091 |
| IB: anti-PTBP1 | This paper | Home-made from ATCC Cat# CRL-2501, RRID: CVCL_L596 |
| IB/IP: anti-PTBP2 | Abcam | Cat# ab57619 RRID: AB_2284865 |
| IB: anti-Lamin B1 | Proteintech | Cat# 66095-1-Ig RRID: AB_2721256 |
| IP: Mouse IgG | Santa Cruz | Cat# sc-2028 RRID: AB_737167 |
| ICC: anti-SOX2 | Stem Cell Technologies | Cat# 60055 RRID: N/A |
| ICC: anti-Tuj1 | Chemicon | Cat# CBL412 RRID: AB_11205398 |
| ICC: anti-Tuj1 | Abcam | Cat# ab107216 RRID: AB_10899689 |
| ICC/IHC: anti-GFP | Abcam | Cat# ab13970 RRID: AB_300798 |
| ICC: anti-SMI-312 | Biolegend | Cat# 837904 RRID: AB_2566782 |
| IHC: anti-Cux1 | Santa Cruz | Cat# sc-13024 RRID: AB_2261231 |
| IHC: FITC donkey anti-chicken | Jackson ImmunoResearch | Cat# 703-095-155 RRID: AB_2340356 |
| IHC: Cy3 donkey anti-mouse | Jackson ImmunoResearch | Cat# 715-165-151 RRID: AB_2315777 |
| IHC: Cy5 donkey anti-rabbit | Jackson ImmunoResearch | Cat# 711-175-152 RRID: AB_2340607 |
| Chemicals, Peptides, and Recombinant Proteins | | |
| Coomassie brilliant blue R-250 | Sangon Biotech | Cat# A602151 |
| Paraformaldehyde | Sigma-Aldrich | Cat# P6148 |
| TRIzol | Invitrogen | Cat# 15596018 |
| DAPI | Sigma | Cat# D9542 |
| Anti-Flag M2 affinity gel | Sigma | Cat# A2220 |
| 3× Flag peptides | APExBio | Cat# A6001 |
| Protease K | Sigma | Cat# P6566 |
| Protein G beads | Roche | Cat# 1243233 |
| Streptavidin magnetic beads | Thermo Scientific | Cat# 88817 |
| Critical Commercial Assays | | |
| KOD-plus mutagenesis kit | Toyobo | Cat# SMK-101 |
| SuperScript II reverse transcriptase | Thermo Fisher Scientific | Cat# 18064014 |
| Lipofectamine 2000 reagent | Invitrogen | Cat# 11668019 |
| Lipofectamine RNAiMAX reagent | Invitrogen | Cat# 13778150 |
| SYBR® Premix Ex Taq™ | Takara | Cat# RR420A |
| Bac-to-Bac® TOPO® expression system | Invitrogen | Cat# A11099 |
| Dual-luciferase reporter assay system | Promega | Cat# E1910 |
| Pierce magnetic RNA-protein pull-down kit | Thermo Scientific | Cat# 20164 |
| Deposited Data | | |
| Single-RNA sequencing data | (*Zhong et al.*, *2018*) | GEO: GSE104276 |
| Whole genome sequencing data | This paper | CNGB: CNP0000742 |
| Experimental Models: Cell Lines | | |
| HEK293 cells | Cell Bank of the Chinese Academy of Sciences (Shanghai, China) | Cat# GNHu18  RRID:CVCL_0045 |
| Sf9 cells | Gibco | Cat# 11496015 |
| WT iPSC | South China Stem Cell Bank (Guangzhou, China) (*Xue et al.*, *2013*) | Cell code #UC-013 |
| Experimental Models: Organisms/Strains | | |
| Mouse: C57BL/6J | Shanghai Laboratory Animal Center, Chinese Academy of Sciences (Shanghai, China) | RRID:IMSR_JAX:000664 |
| Mouse: *Fgf13* Mutant | This paper | Home-made |
| Bacteria: DH10Bac | Gibco | Cat# 10361012 |
| Oligonucleotides | | |
| Primers for plasmid, oligomer and PCR | see Table S5 | N/A |
| Recombinant DNA | | |
| pcDNA3.1/myc-his(-) vector | Invitrogen | Cat# V855-20 |
| pcDNA3.1-WT-hFGF13-TV2 + CAG-HA-RFP | This paper | N/A |
| pcDNA3.1-Mut-hFGF13-TV2 + CAG-HA-RFP | This paper | N/A |
| pcDNA3.1-WT-hFGF13-TV4 + CAG-HA-RFP | This paper | N/A |
| pcDNA3.1-Mut-hFGF13-TV4 + CAG-HA-RFP | This paper | N/A |
| pcDNA3.1-WT-mFGF13-TV2 + CAG-HA-RFP | This paper | N/A |
| pcDNA3.1-Mut-mFGF13-TV2 + CAG-HA-RFP | This paper | N/A |
| pcDNA3.1-WT-mFGF13-TV4 + CAG-HA-RFP | This paper | N/A |
| pcDNA3.1-Mut-mFGF13-TV4 + CAG-HA-RFP | This paper | N/A |
| psiCHECK^TM^-WT-hFGF13-5'-UTR-Rluc | This paper | N/A |
| psiCHECK^TM^-Mut-hFGF13-5'-UTR-Rluc | This paper | N/A |
| psiCHECK^TM^-WT-mFGF13-5'-UTR-Rluc | This paper | N/A |
| psiCHECK^TM^-Mut-mFGF13-5'-UTR-Rluc | This paper | N/A |
| PX459-WT-hFGF13 sgRNA | This paper | N/A |
| PX459-Mut-hFGF13 sgRNA | This paper | N/A |
| PX459-mFGF13 sgRNA | This paper | N/A |
| pCAG-YFP | This paper | Addgene# 11180 |
| pcDNA3-HA-RPL22 | (*Sanz et al.*, *2009*) | N/A |
| pRIF-Rluc-ECMV IRES-Firefly | Gift from Dr. Ligang Wu | N/A |
| pRIF-Rluc-WT-hFGF13-5'-UTR- Firefly | This paper | N/A |
| pRIF-Rluc-Mut-hFGF13-5'-UTR- Firefly | This paper | N/A |
| pRIF-Rluc-Random sequence- Firefly | This paper | N/A |
| pFastBac1-Flag-hPTBP1 | This paper | N/A |
| pFastBac1-Flag-hPTBP2 | This paper | N/A |
| pcDNA3-Flag-hPTBP2 | This paper | N/A |
| pcDNA3-Flag-MCP-hPTBP2 | This paper | N/A |
| pcDNA3-MS2-hFGF13-TV2 | This paper | N/A |
| pcDNA3-Flag-PUF-Vector | This paper | N/A |
| pcDNA3-Flag-wPUF-mPTBP2 | This paper | N/A |
| pcDNA3-Flag-mPUF-mPTBP2 | This paper | N/A |
| pCDH-Flag-PUF-Vector | This paper | N/A |
| pCDH-Flag-wPUF-mPTBP2 | This paper | N/A |
| pCDH-Flag-mPUF-mPTBP2 | This paper | N/A |
| Software and Algorithms | | |
| GraphPad Prism | GraphPad Software | <http://www.graphpad.com/scientificsoftware/prism/>, RRID:SCR_002798 |
| ImageJ | National Institutes of Health | <https://imagej.nih.gov/ij/>, RRID:SCR_003070 |
| Others | | |
| Amaxa Nucleofector II System | Lonza Amaxa, Germany | https://bioscience.lonza.com/lonza_bs/CH/en/Transfection/p/000000000000199466/Nucleofector-2b-Device |
| GloMax® 20/20 Luminometer | Promega | https://www.promega.com.cn/products/microplate-readers-fluorometers-luminometers/microplate-luminometers/glomax-20_20-luminometer/?catNum=E5311 |
| ECM-830 BTX square wave electroporator | VWR International | https://us.vwr.com/store/product/4694246/ecm-830-square-wave-electroporation-system-btxtm |

*Note: IB/IP/IHC/ICC are short for immunoblotting, immunoprecipitation, immunohistochemistry and immunocytochemistry, respectively.*
